# Comparing pseudo-absences generation techniques in Boosted Regression Trees models for conservation purposes: A case study on amphibians in a protected area

**DOI:** 10.1371/journal.pone.0187589

**Published:** 2017-11-06

**Authors:** Francesco Cerasoli, Mattia Iannella, Paola D’Alessandro, Maurizio Biondi

**Affiliations:** Department of Life, Health & Environmental Sciences, University of L’Aquila, L’Aquila, Italy; Imperial College Faculty of Medicine, UNITED KINGDOM

## Abstract

Boosted Regression Trees (BRT) is one of the modelling techniques most recently applied to biodiversity conservation and it can be implemented with presence-only data through the generation of artificial absences (pseudo-absences). In this paper, three pseudo-absences generation techniques are compared, namely the generation of pseudo-absences within target-group background (TGB), testing both the weighted (WTGB) and unweighted (UTGB) scheme, and the generation at random (RDM), evaluating their performance and applicability in distribution modelling and species conservation. The choice of the target group fell on amphibians, because of their rapid decline worldwide and the frequent lack of guidelines for conservation strategies and regional-scale planning, which instead could be provided through an appropriate implementation of SDMs. *Bufo bufo*, *Salamandrina perspicillata* and *Triturus carnifex* were considered as target species, in order to perform our analysis with species having different ecological and distributional characteristics. The study area is the “Gran Sasso—Monti della Laga” National Park, which hosts 15 Natura 2000 sites and represents one of the most important biodiversity hotspots in Europe. Our results show that the model calibration ameliorates when using the target-group based pseudo-absences compared to the random ones, especially when applying the WTGB. Contrarily, model discrimination did not significantly vary in a consistent way among the three approaches with respect to the tree target species. Both WTGB and RDM clearly isolate the highly contributing variables, supplying many relevant indications for species conservation actions. Moreover, the assessment of pairwise variable interactions and their three-dimensional visualization further increase the amount of useful information for protected areas’ managers. Finally, we suggest the use of RDM as an admissible alternative when it is not possible to individuate a suitable set of species as a representative target-group from which the pseudo-absences can be generated.

## Introduction

In conservation biology, one of the most important activities is monitoring population abundances and distribution, both for animals and plants. Studying changes over time in an animal distribution means understanding its temporal dynamics, also assessing management effectiveness [1]. As reported by Marsh and Trenham [2], there are many different monitoring techniques to evaluate animal population dynamics, and the data obtained can be used to identify endangered species or real and potential diffusion of invasive and pest species [3–6]. An important role in the conservation of populations is played by the protected areas (PAs). Many conservationists celebrate the expansion of protected territories and the increasing attention paid to biodiversity, but they often disagree on how to manage parks and reserves. In recent years, the development of several modelling approaches has allowed ecologists to better understand the potential diffusion of animals and plants and predict their distribution within changing environmental scenarios [7], and this represents a promising tool in biodiversity conservation issues.

During the last decade, the use of different SDMs (Species Distribution Models) to assess the actual and potential distribution of species has gained increasing popularity among ecologists [8–10]. This growing interest in the implementation of ecological modelling within distributional issues arises from both the increasing availability of occurrence data for a huge number of taxa gathered in public institutions and private collections [11–13], and the recent development of new modelling approaches (e.g. Machine Learning) which allow researchers to model complex ecological responses (e.g. [14, 15]).

In recent studies, different modelling techniques have been applied to the same target species, comparing their predictive performance and their capacity to account for complex relationships among the ecological, climatic and spatial predictors which are assumed to have a significant role in shaping the species’ distribution [16–19]. One of the most recently debated issues about ecological modelling applications is how to deal with the “absences” [10, 17, 20–23]. In fact, most of the available data on species’ occurrences are represented by ensembles of presence records, often collected by different researchers in different timeframes, and only for a relatively small number of species systematic surveys have been carried out, so as to permit a reliable assessment of the presence and absence sites within a certain area. Some approaches permitting to build presence-absence models using presence data sets through the generation of artificial absence data, referred to as ‘pseudo-absences’, were recently introduced in machine-learning techniques [19, 24, 25].

Among them, Boosted Regression Trees (BRT) [15, 16] is one of the modelling techniques most recently applied to conservation issues (e.g. [24, 26–28]). BRT results from the combination of regression trees, which belong to the decision tree group of models, and boosting technique, which allows modellers to produce a large number of simple tree models and then combine them so as to optimize predictive potential [15]. The BRT technique is capable of properly fitting complex functions, which reflect the complexity of the ecological processes shaping the species’ distribution; it can select the most relevant variables within the set of input predictors and model the interactions among them, if present [15]. Furthermore, BRT can handle categorical as well as continuous input variables, it includes algorithms which allow the creation of a large number of models from which a final optimized model can be obtained, and it has been reported to assure good performances with a low to moderate number of occurrence data [15, 17]. Several approaches to generate pseudo-absences have been tested in recent years [19, 24]. In the present study we focused on three of them within the BRT modelling technique and compared their predictive performance and robustness on three amphibian species, differing in their distribution and ecological requirements (see Materials and methods). In addition, we provide suggestions on how to deal with the pseudo-absences when modelling the distribution of species needing conservation actions. Notwithstanding several studies using simulated species to test metrics for model validation have been published recently (e.g. [20, 29, 30]), we intentionally chose to use a database referring to reliably assessed presences for the species considered, in order to address more specifically the potentialities and drawbacks that conservationists should consider when applying the SDM approach to biodiversity conservation and reserve planning.

For this aim, a target group comprising three amphibian species occurring in Central Apennines, having different distributional and ecological characteristics, was considered for our analyses: *Salamandrina perspicillata* (Savi, 1821) and *Triturus carnifex* (Laurenti, 1768), both endemic to the peninsular Italy; *Bufo bufo* (Linnaeus, 1758), widespread across Europe. Amphibians represent, in fact, a taxon that often need a better integrated approach for conservation actions, since this class of vertebrates is undergoing a rapid decline with high rates of species loss [31]. Finally, amphibians are rarely well-represented in conservation plans [32] and suffer from “high-level” studies, resulting in little research and few guidelines on practical conservation measures [33], notwithstanding their undoubted ecological role.

## Materials and methods

### Target species and study area

Three target species were considered for our research:

- *Bufo bufo* (Linnaeus, 1758), considered “Vulnerable” in IUCN Italy Red List [34], is a generalist species, widely distributed across Europe and part of Asia and North Africa;
- *Salamandrina perspicillata* (Savi, 1821), included in Annex II of the EU Habitats Directive and considered “Least Concern” in IUCN Italy Red List [35], is an endemic salamander of Apennines, particularly linked to intact woodland habitats and streams;
- *Triturus carnifex* (Laurenti, 1768), included in Annex II of the EU Habitats Directive and considered “Near Threatened” in IUCN Italy Red List [36], is an endemic newt of peninsular Italy related to lentic ecosystems.

**Fig 1:**
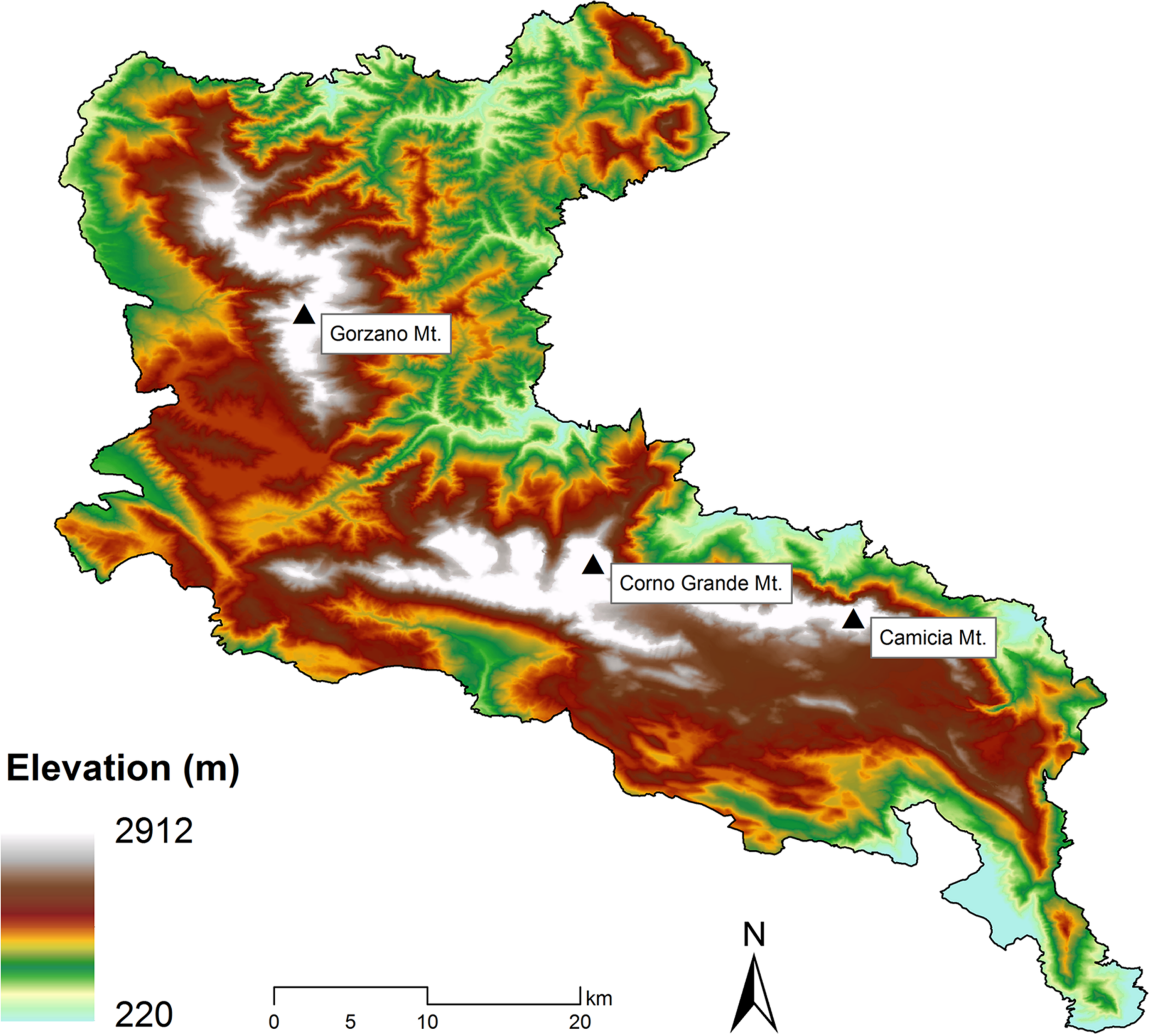
Study area. “Gran Sasso-Monti della Laga” (GSML) National Park. The highest peaks of the main districts are indicated by the black triangles.

Presence records for each species and target-group background data [25] (S1 Dataset) were derived from the database generated in Iannella [37]. The study area includes the “Gran Sasso-Monti della Laga” National Park (GSML National Park) (Fig 1), a 1.432 km^2^-wide PA in the central Apennines (Italy), which hosts 15 “Natura 2000” sites (ftp://ftp.minambiente.it/PNM/Natura2000/.TrasmissioneCE_2015/).

The choice of the study area was based on both the availability of reliable data and the intent of our analysis to investigate the applicability of SDMs to conservation issues (e.g. management of PAs). Indeed, an extensive survey on the whole GSML National Park territory was carried out by specialists in 2013, funded in the context of the EU project “Natura 2000”; the resulting data on all the amphibian presence sites in the Park are comprised in Iannella [37]. The presence data used in this study represent therefore reliable records at GPS resolution, and allow to assume detectability as constant across all the dataset [20]. For each species, only presence data falling within the study area were used, since the use of presence data from other areas and the following projection of the resulting model to a target study area may generate greater uncertainties [38].

Spatial autocorrelation within presence records of each species and within the aggregated presence data was tested using a 1 × 1 km grid, derived from the 10 × 10 km UTM grid, through Moran’s I test. The 1 × 1 km grid was chosen in order to avoid the loss of any relevant fine-scale spatial and environmental information available in the dataset used. Each record was reported in geographical coordinates (UTM-WGS84 reference system); spatial analysis and distribution maps were generated with ESRI ArcGis 10.0 software.

### Environmental variables

The set of environmental predictors used comprises: a) nineteen bioclimatic variables (BIO1-BIO19) [39] and b) elevation data (ALT), downloaded from the Worldclim database (http://www.worldclim.com/current/); c) two topographic variables, namely SLOPE, representing the incline of the surface, and ASPECT, representing the “exposure”, which is the compass direction that a topographic slope faces [40]. The latter two variables are expressed in degrees and were derived from a Digital Elevation Model originating from the elevation data, using the “surface tool” in ArcGis Spatial Analyst. All these variables were used with a spatial resolution of 30 arc-seconds (0.93 x 0.93 = 0.86 km^2^ at the equator), in conformity with the high precision of the spatial information contained in the presence dataset.

### Model building

The Boosted Regression Trees (BRT) technique was implemented in our study in three different ways:

1. pseudo-absences for each target species established on the presence points of the other amphibian species occurring in the study area, thus setting up a “non-overlapping target-group background” [25]. Using this approach, hereafter named UTGB (unweighted target-group background), to select pseudo-absence points, eventual bias in sampling design would be similar for all the target species, and this may produce better modelling results [25].
2. pseudo-absences for each target species established on the presence points of the other amphibian species occurring in the study area (see above), implementing a weighting scheme so that the sum of the weights on the pseudo-absences equals the sum of those on the presence points [24, 41]. Assuming that *D* is the total number of presence points in the dataset, *p*_*k*_ = total of the *n*-presence points of *k*-species, *a*_*k*_ = number of target-group pseudo- absences for the *k*-species, then *a*_*k*_ = *D—p*_*k*_; therefore, the weight *W*_*ik*_ of the *i*-pseudo-absence point of the *k*-species will be *W*_*ik*_ = *p*_*k*_ */ a*_*k*_, so that ∑*W*_*ik*_ × *a*_*ik*_ *= p*_*k*_.
  This approach is hereafter named WTGB (weighted target-group background).
3. pseudo-absences generated through random selection of points from the whole study area except the occurrence localities of the target species, selecting a number of random pseudo-absences equal to the number of presence records, since this ratio between presences and pseudo-absences seems to assure good accuracy in BRT models [24]. Four replicates of the random pseudo-absences generation process, hereafter RDM, were performed, and predictions from the corresponding models were then averaged.

A total of 320 amphibians’ presence records formed the starting data set. For each target species, Table 1 shows the number of presence records and the number of pseudo-absences used to build models through the UTGB, WTGB and RDM approaches.

**Table 1:** Presence and pseudo-absence data for the three target species.

Species	Presence data	Pseudo-absences	
		UTGB and WTGB*	RDM
*Bufo bufo*	73	247	73
*Salamandrina perspicillata*	24	296	24
*Triturus carnifex*	55	265	55

For each target species are indicated the number of presence data and the number of pseudo-absences used within the three different implementations of the Boosted Regression Trees model.

*UTGB and WTGB were aggregated in a single column since for both methods the number of pseudo-absences of the *k*-species is *a*_*k*_ *= D—p*_*k*_, with D total number of occurrences in the dataset and *p*_*k*_ number of occurrences of the *k*-species

in WTGB each pseudo-absence has a weight *W*_*ik*_ *= p*_*k*_ */ a*_*k*_.

All the BRT models were built in R [42] version 3.2.3 (https://cran.r-project.org/bin/windows/ base/old/3.2.3/) using the package “gbm” version 2.1.1 [43] and the additional function “gbm.step” provided by J. Elith and J.R. Leathwick in Elith, Leathwick [15]. In BRT, the regularization parameters which most influence variable selection and model performance are mainly three: a) the shrinkage parameter, also named “learning rate”, limiting the contribution of the single trees which are added in sequence to the model through the boosting algorithm; b) the “tree complexity”, indicating the order of the interactions between the predictors that would be modelled, so that if tree complexity equals 2 a model with up to two-way interactions will be built. Learning rate and tree complexity influence the number of trees required to reach the optimal model performance [15]; c) the “bag fraction”, representing the proportion of data which are randomly drawn without replacement from the full data set at each iteration and used to build the current model; this parameter introduces stochasticity in BRT model building, thus reducing overfitting and improving accuracy [15, 44].

BRT models were produced using the following parameterization setting: learning rate = 0.001 in order to allow each model to reach at least 1000 trees, following a “rule of thumb” for balancing model complexity and performance [15, 45]; tree complexity = 5; bag fraction = 0.5 or 0.75, depending on which one results in the best model performance for each single species.

### Model evaluation

The debate on which methods are appropriate to evaluate the performance of SDMs, particularly when dealing with models built on presence-background data [20, 21], is ongoing and still controversial, since there is lack of clear guidelines to test different aspects of the modelling outcomes [22]. In this paper, discrimination capability of the models obtained was evaluated by means of two metrics, the AUC, namely the area under the receiver operating characteristic (ROC) curve [46], and the TSS (i.e. True Skill Statistic) [47]. AUC is a threshold-independent statistic used in ecological modelling to assess the capability of a model to discriminate between positive (presences) and negative (absences) instances [14, 17]. Even though the use of AUC to evaluate discrimination performance of SDMs has been questioned [29, 48], this metric is used in several recent papers, dealing with biodiversity conservation (e.g. [26–28]) or other research fields [49, 50], even when the authors themselves state that the absence data they use may not reflect real absences [28]. We used AUC as discrimination metric for the BRT models obtained because, even though it should not be used to compare models for different species in presence-background models [29], it can still be used to compare different modelling approaches applied to the same species at the same extent [48], which is one of the main purposes of this paper. The function “gbm.step” allowed us to calculate the AUC score and the deviance, namely a loss function used in BRT models to measure the loss in predictive performance consequent on suboptimal models [15], within a 10-fold cross-validation framework. More generally, deviance represents a measure of calibration for models with continuous outputs [51].

Statistical significance of differences in the AUC and deviance values resulting from the cross-validated BRT models was tested for each target species and each pseudo-absence generation approach. With regard to UTGB and WTGB, we considered the values of AUC and deviance within each of the ten cross-validation folds in the final optimized model (*n* = 10 for each metric for both methods), while for the RDM approach we considered the mean cross-validated AUC and deviance values for each of the four replicates performed (*n* = 4 for each metric). First, we evaluated normality (intra-species, intra-model) through Shapiro-Wilk test and homoscedasticity (intra-species, inter-model) through Levene’s test. When both normality and homoscedasticity were confirmed, we performed two-tailed *t*-tests between each pair of modelling approaches for a same species, in order to assess possible significant differences in AUC and/or deviance among UTGB, WTGB and RDM. When normality or homoscedasticity were not verified, we performed the non-parametrical Wilcoxon-Mann-Whitney tests for the same purpose. The results of all the tests were evaluated considering a critical *p*-value of 0.05.

TSS, instead, defined as sensitivity+specificity-1, is a threshold-dependent measure of discrimination widely used for SDMs resulting in binary presence-absence outputs [47, 51, 52]. Therefore, it is particularly useful for the application of SDMs to spatial prioritization of areas for conservation measures, since reserve managers often prefer a binary representation of the areas predicted as suitable or not for the target species, even though Guillera-Arroita et al. (2015) [20] showed that binary discretization of the continuous output of SDMs often reduce their predictive power. We used the R package “ecospat” version 2.1.1 [53] to calculate the TSS, for each target species and each pseudo-absence generation approach, along increasing threshold values (increment of 0.01 each time), comparing the binary presence-pseudoabsence values of each target species with the predictions resulting from the corresponding BRT model. Once calculated the TSS along increasing threshold values, we reported the values of TSS for two of the thresholds most widely used to convert continuous model outputs into binary ones, namely the threshold that maximises TSS and the one corresponding to the 10^th^ percentile estimate for training presences [20, 52, 54].

Maps representing the modelled distribution of the target species were generated from the BRT projections over the whole study area in raster format, indicating the relative likelihood of occurrence (output of a presence-background model built on unbiased data characterized by constant detectability [20]) at each pixel within a continuous scale 0–1. Within the RDM approach, the raster outputs resulting from the four replicates were averaged. In order to better compare the models resulting from the different approaches, the continuous raster outputs were made discrete by considering 5 equally distributed classes of increasing relative likelihood of occurrence (i.e. 0–0.2; 0.2–0.4; 0.4–0.6; 0.6–0.8; 0.8–1.0), and then areas for each class were calculated in ArcMap 10.0.

Relative contributions, expressed as percentages and computed considering the number of times a certain variable is selected for splitting during tree building, weighted by the consequent squared improvement in the model and averaged over all trees [15], and pairwise interaction sizes, indicating the effect of two predictors interacting each other on the response variable [15], were assessed for each BRT model. Finally, partial dependence plots, which report the variation of relative likelihood of occurrence consequent upon the variation in the selected predictors, were generated using SYSTAT 13 statistical software for the three predictors showing the highest relative contributions.

## Results

Presence data for each of the three target species showed no spatial autocorrelation (Moran’s I = 0.108, p-value = 0.161, z-score = 1.402 for *B*. *bufo*, Moran’s I = 0.069, p-value = 0.219, z-score = 1.228 for *S*. *perspicillata* and Moran’s I = -0.200, p-value = 0.314, z-score = -1.007 for *T*. *carnifex*); the absence of spatial autocorrelation was confirmed also when the whole dataset was tested (Moran’s I = 0.044, p-value = 0.162, z-score = 1.395).

The values of the cross-validated AUC and deviance resulting from each BRT model are reported in Table 2.

**Table 2:** AUC and deviance for each pseudo-absence generation method and each target species.

Species	UTGB		WTGB		Averaged RDM	
	cv AUC ± SE	cv deviance ± SE	cv AUC ± SE	cv deviance ± SE	mean cv AUC ± SE	mean cv deviance ± SE
*Bufo bufo*	0.646 ± 0.035	1.036 ± 0.026	0.631 ± 0.035	0.621 ± 0.010	0.729 ± 0.026	1.234 ± 0.041
*Salamandrina perspicillata*	0.833 ± 0.043	0.426 ± 0.051	0.882 ± 0.027	0.166 ± 0.019	0.854 ± 0.057	0.920 ± 0.181
*Triturus carnifex*	0.783 ± 0.035	0.784 ± 0.034	0.757 ± 0.047	0.409 ± 0.019	0.899 ± 0.021	0.831 ± 0.092

Values of the cross-validated AUC and deviance, together with their respective standard errors, are shown for each species and each pseudo-absences generation approach.

*B*. *bufo* shows AUC scores lower than 0.7 in both UTGB and WTGB, with only a slight improvement in the RDM approach. *S*. *perspicillata* attains AUC scores higher than 0.8 in all the three pseudo-absences generation approaches, reaching the maximum score in WTGB. Finally, *T*. *carnifex* shows AUC scores higher than 0.7 in UTGB and WTGB, but there is an apparent increase of discrimination within the RDM approach, with an AUC score of approximately 0.9.

Considering the cross-validated deviance, all the three species show a noticeable reduction of deviance in WTGB with respect to the UTGB and RDM, with the latter approach always resulting in the highest deviance values.

The Shapiro-Wilk tests confirmed the null hypothesis of normality for all the ‘intra-species intra-model’ samples (*p* > 0.05) for both AUC and deviance. The Levene’s tests performed considering the AUC confirmed the null hypothesis of homoscedasticity for all the ‘intra-species inter-model’ samples (*p* > 0.05); with regard to deviance, the null hypothesis of homoscedasticity was rejected for *T*. *carnifex* and *S*. *perspicillata* (*W* = 6.595, *p* = 0.0059 for *T*. *carnifex*; *W* = 12.22, *p* = 0.0003 for *S*. *perspicillata*), while it was confirmed for *B*. *bufo* (*W* = 2.6633, *p* = 0.09).

Since both normality and homoscedasticity were confirmed for all the AUC samples, we performed the two-tailed *t*-tests for each possible pair of pseudo-absence generation methods for each target species, resulting in no statistically significant difference in AUC (*p* > 0.05) for all pairs except RDM-WTGB for *B*. *bufo* (*t* = 2.251, *p* = 0.0451), RDM-UTGB for *T*. *carnifex* (*t* = 2.824, *p* = 0.0154) and RDM-WTGB for *T*. *carnifex* (*t* = 3.284, *p* = 0.0097). The two-tailed *t*-tests performed for *B*. *bufo*, (since both normality and homoscedasticity were verified) considering the deviance values resulted in statistically significant differences among all the three modelling approaches (UTGB-WTGB, *t* = 14.774, *p* = 7.06e^-9^; UTGB-RDM, *t* = -4.0691, *p* = 0.0075; WTGB-RDM, *t* = -14.547, *p* = 0.0004). For *S*. *perspicillata* and *T*. *carnifex*, instead, since homoscedasticity was not confirmed by the Levene’s tests, we performed the Wilcoxon-Mann-Whitney tests. Significant differences in deviance among the three pseudo-absence generation techniques were confirmed for both *S*. *perspicillata* (UTGB-WTGB, *W* = 99, *p* = 2.16e^-5^; UTGB-RDM, *W* = 3, *p* = 0.014; WTGB-RDM, *W* = 0, *p* = 0.002) and *T*. *carnifex* (UTGB-WTGB, *W* = 100, *p* = 0.0002; WTGB-RDM, *W* = 0, *p* = 0.0058), with only the pair UTGB-RDM for *T*. *carnifex* showing no significant difference (*W* = 16, *p* = 0.635).

The values of TSS and the corresponding thresholds for the two threshold-selection methods considered (see Materials and methods) are shown in Table 3, while plots showing the TSS variation in response to different thresholds are reported in S2 Fig and S3 Fig.

**Table 3:** Values of the TSS and the corresponding thresholds resulting from the two threshold-selection methods ‘10^th^ percentile estimate for training presences’ and ‘TSS max’.

	**TSS 10th percentile**					
	**UTGB**		**WTGB**		**RDM**	
	Threshold	TSS	Threshold	TSS	Threshold	TSS
*Bufo bufo*	0.19116	0.47254	0.4352	0.4476	0.51926	0.79862
*Salamandrina perspicillata*	0.14411	0.81696	0.49443	0.80465	0.67554	0.77422
*Triturus carnifex*	0.17475	0.65317	0.45433	0.62554	0.81107	0.84283
	**TSS max**					
	**UTGB**		**WTGB**		**RDM**	
*Bufo bufo*	0.25	0.568	0.47	0.5597	0.48749	0.81532
*Salamandrina perspicillata*	0.12	0.90878	0.45	0.89527	0.41341	0.8826
*Triturus carnifex*	0.17	0.65317	0.41	0.63911	0.57357	0.96877

For each target species and each pseudo-absences generation approach are shown the values of the TSS, corresponding to the two threshold-selection methods ‘10^th^ percentile estimate for training presences’ and ‘TSS max’, and the relative thresholds.

**Fig 2:**
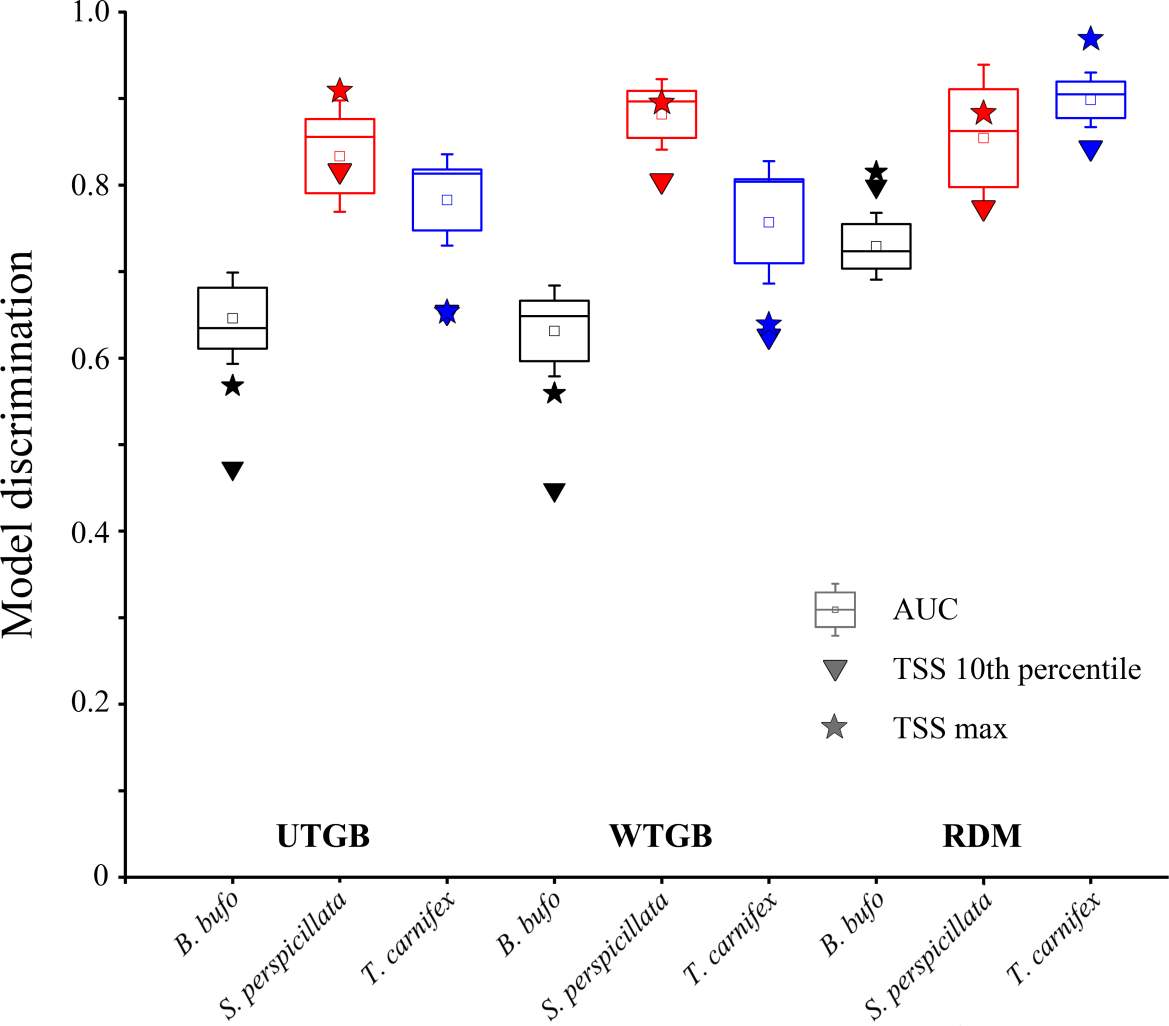
Boxplots representing the AUC scores for each target species within the three modelling approaches tested. For UTGB and WTGB, boxplots were built considering the AUC values from each of the ten cross-validation folds in the final optimized model, while for RDM boxplots were built considering the mean cross-validated AUC values resulting from each of the four replicates performed. The small squares within the boxes represent the mean AUC value, the horizontal bars within the boxes represent the median and the whiskers represent the standard error. Triangles represent the values of TSS at 10^th^ percentile, while stars represent the value of the maximum TSS.

**Fig 3:**
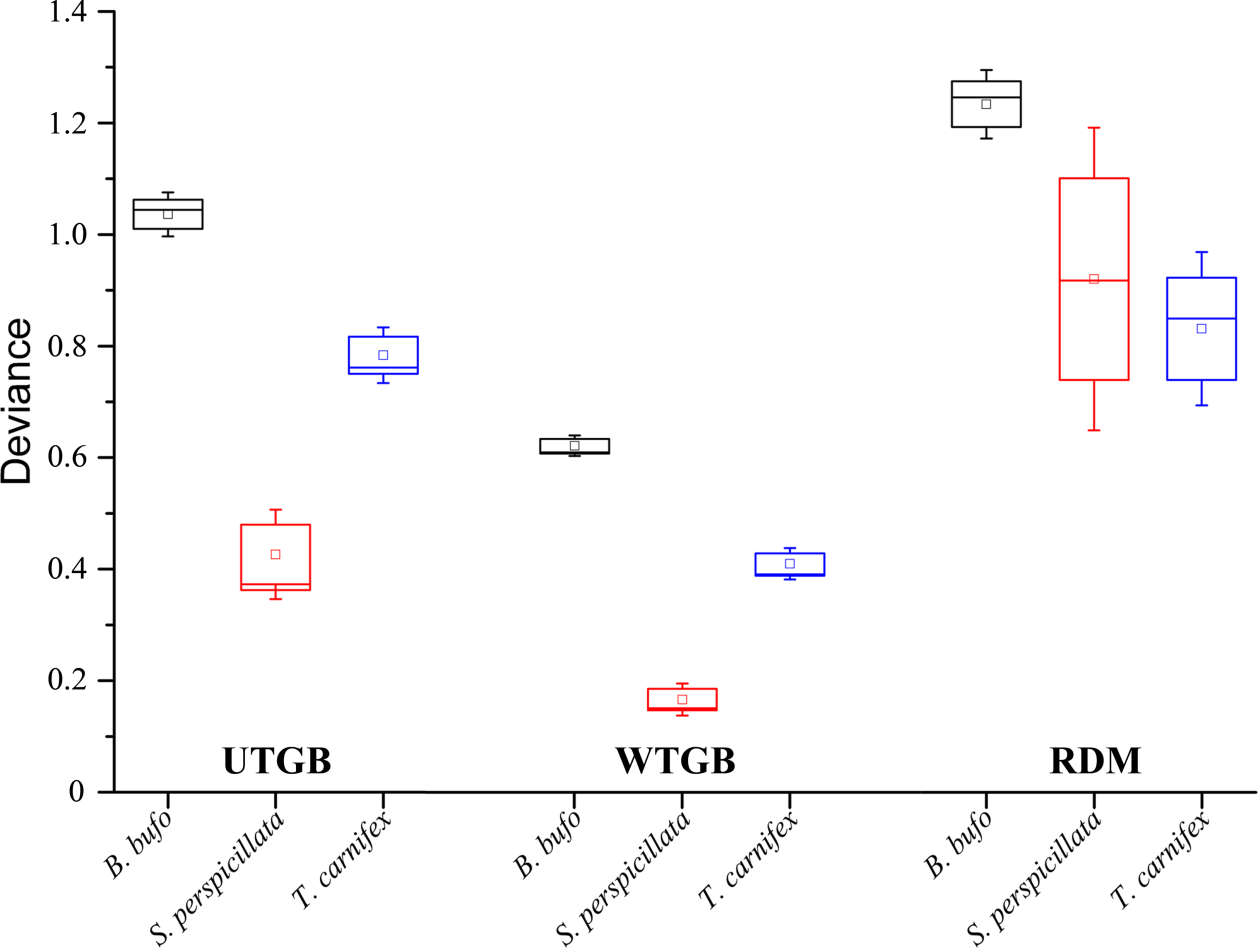
Boxplots representing the deviance scores for each target species within the three modelling approaches tested. For UTGB and WTGB, boxplots were built considering the deviance values from each of the ten cross-validation folds in the final optimized model, while for RDM boxplots were built considering the mean cross-validated deviance values resulting from each of the four replicates performed. The small squares within the boxes represent the mean deviance value, the horizontal bars within the boxes represent the median and the whiskers represent the standard error.

The boxplots in Fig 2 represent the scores of AUC, TSS ‘10^th^ percentile’ and TSS max for each species and each pseudo-absence generation approach, while those in Fig 3 result from the corresponding deviance values.

**Fig 4:**
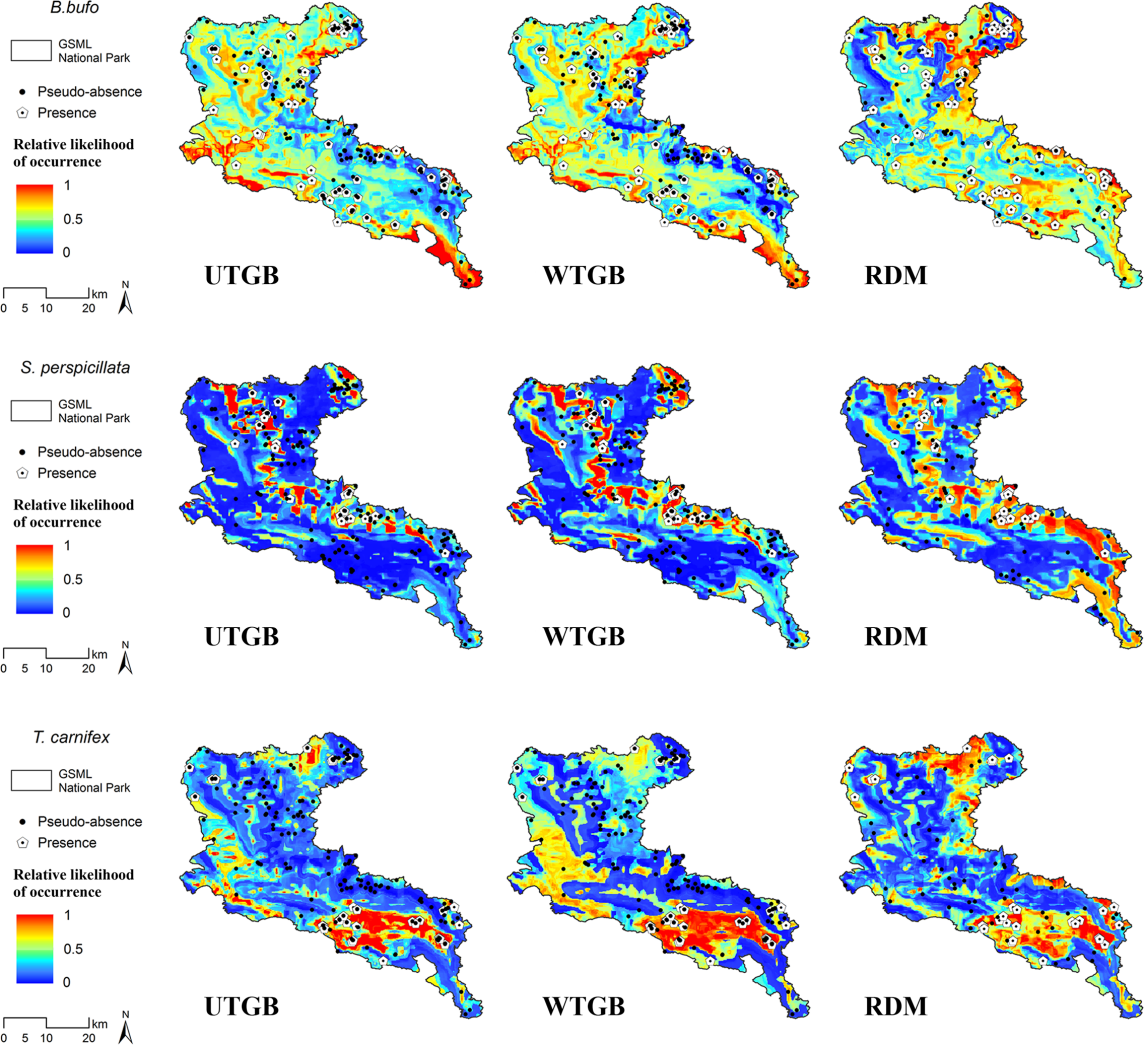
Modelled distribution of each target species. Maps result from (left to right): 10-fold cross-validated Boosted Regression Trees (BRT) model built on non-overlapping target-group background (UTGB); 10-fold cross-validated BRT model built on weighted non-overlapping target-group background (WTGB); average over 4 replicates of 10-fold cross-validated BRT model built on pseudo-absences drawn at random excluding presence localities (RDM). Within the maps resulting from RDM, the shown pseudo-absences were taken from one of the replicates, only for graphical purposes.

In Fig 4 are shown, for each target species, the modelled distributions obtained from UTGB, WTGB and RDM; the latter represents the distribution averaged over the four replicates performed, while the single maps for each replicate are shown in S1 Fig. Relative likelihood of occurrence is reported, as default, in a continuous format, with increase in the likelihood values represented through a blue-to-red scale. In Table 4, instead, the extent of the areas corresponding to each of the five classes of relative likelihood of occurrence is reported for each species. Some interesting patterns emerge considering Fig 4 and Table 4. The models built on the UTGB show for all the target species a clear predominance of areas included in the classes 0–0.2 and 0.2–0.4, especially for *S*. *perspicillata*, whose modelled distribution is mainly restricted to areas surrounding the known occurrence localities. On the contrary, the models built through the WTGB revealed an increase in the extent of areas corresponding to the three central classes (from 0.2–0.4 to 0.6–0.8). As well as for WTGB, predictions resulting from the averaged RDM models are clearly more shifted towards the three central classes with respect to the UTGB models. In RDM models, the extent of areas corresponding to the two upper classes (0.6–0.8 and 0.8–1.0) is higher than that resulting from WTGB for all the three target species, even though the class 0.8–1.0 shows low percentages over the total area. The averaged RDM covers all the five classes, still returning higher percentages for the low-middle classes (from 0–0.2 to 0.4–0.6).

**Table 4:** Extent of predicted areas for the three species and three methods tested.

Species	Relative likelihood of occurrence	UTGB		WTGB		RDM (AVG)	
		Extent (ha)	Percentage on total area	Extent (ha)	Percentage on total area	Extent (ha)	Percentage on total area
*Bufo bufo*	0–0.2	37,838	26.42	0	0.00	4,355	3.04
	0.2–0.4	100,890	70.46	13,745	9.60	45,924	32.07
	0.4–0.6	4,464	3.12	129,148	90.19	67,202	46.93
	0.6–0.8	0	0.00	298	0.21	24,811	17.33
	0.8–1	0	0.00	0	0.00	900	0.63
*Salamandrina perspicillata*	0–0.2	131,910	92.12	54,715	38.21	56,955	39.78
	0.2–0.4	8,477	5.92	61,866	43.21	30,328	21.18
	0.4–0.6	2,326	1.62	19,507	13.62	20,815	14.54
	0.6–0.8	478	0.33	7,102	4.96	23,812	16.63
	0.8–1	0	0.00	0	0.00	11,282	7.88
*Triturus carnifex*	0–0.2	116,843	81.60	54,199	37.85	70,662	49.35
	0.2–0.4	23,264	16.25	41,928	29.28	25,391	17.73
	0.4–0.6	3,065	2.14	35,718	24.94	23,173	16.18
	0.6–0.8	19	0.01	11,347	7.92	17,572	12.27
	0.8–1	0	0.00	0	0.00	6,394	4.47

Extent and corresponding percentage of the total study area for each class of relative likelihood of occurrence, as result in the BRT models obtained from the three pseudo-absence generation approaches compared. Areas are reported in hectares.

Table 5 reports, for each target species and for each modelling approach tested, the three variables showing the highest relative contribution scores. Overall, topographic and precipitation-related predictors seem to primarily influence the models for all the three target species. Nonetheless, the three approaches tested show some differences in the combinations of most influential predictors and in the contribution scores of these latter, particularly for *B*. *bufo*. In fact, it presents the most changeable range of influential predictors: even though ASPECT resulted as the most contributing variable in all the three approaches tested, its contribution score is not relevantly higher than the others, and the second and third most influential predictors are different among the three approaches.

**Table 5:** Variables contribution for the three species and three methods tested.

Species	Relative contribution					
	UTGB		WTGB		Averaged RDM	
	Variable	Score (%)	Variable	Score (%)	Variable	Score (%)
*Bufo bufo*	ASPECT	13.76	ASPECT	17.50	ASPECT	13.40
	BIO8	12.23	BIO8	12.36	BIO13	11.89
	BIO14	9.59	BIO4	9.78	BIO17	11.18
*Salamandrina perspicillata*	SLOPE	19.89	SLOPE	36.76	SLOPE	41.31
	ASPECT	19.74	ASPECT	19.79	ASPECT	26.76
	BIO12	13.50	BIO12	12.59	BIO12	5.39
*Triturus carnifex*	SLOPE	33.13	SLOPE	43.98	SLOPE	24.27
	BIO12	10.80	BIO12	9.80	BIO12	13.51
	ASPECT	7.33	ASPECT	7.37	BIO16	9.47

For each target species are reported the three variables showing the highest relative contribution within the BRT models resulting from the three modelling approaches compared, and their corresponding percentage scores.

**Fig 5:**
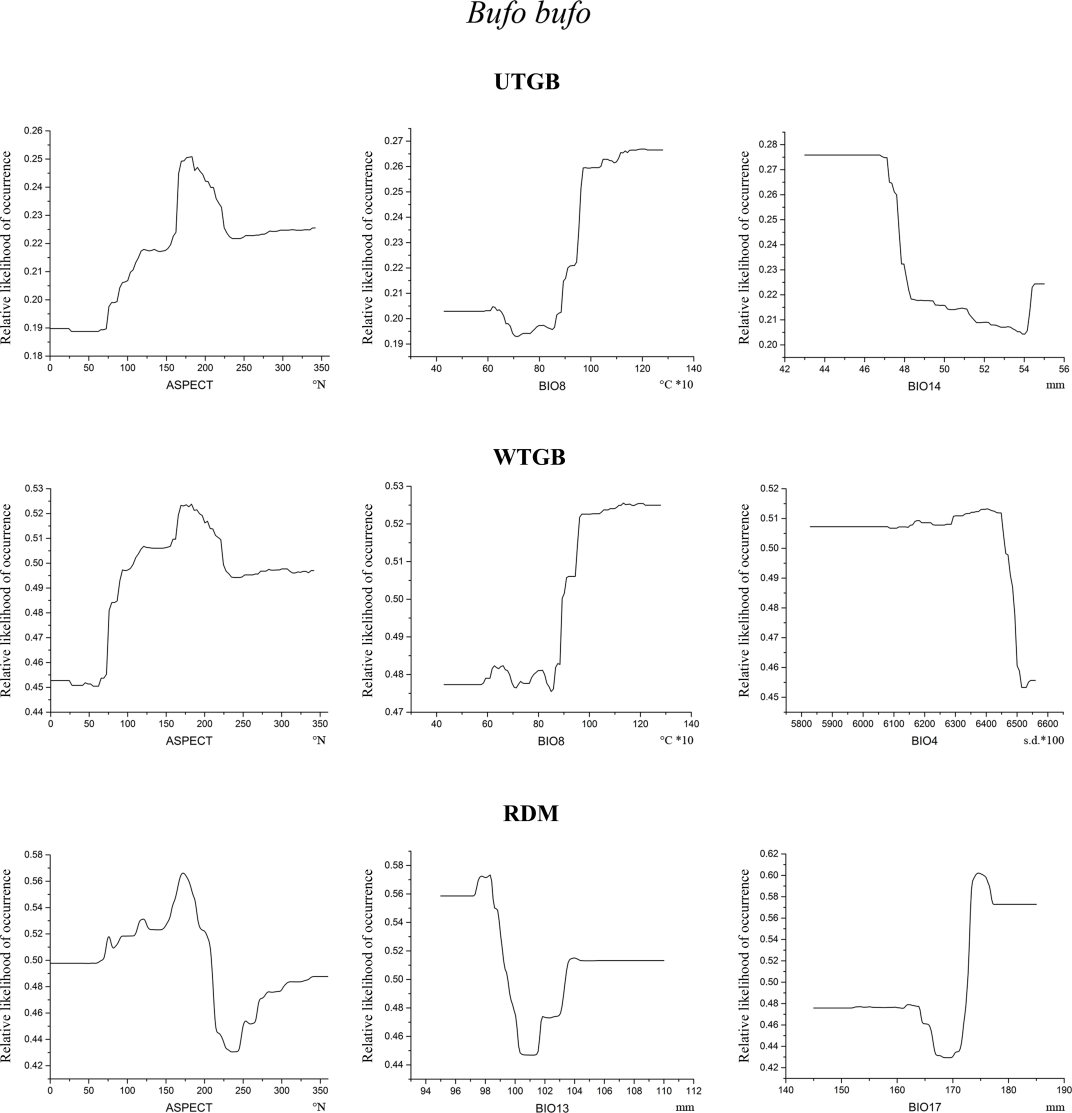
Partial dependence plots for *B*. *bufo*. Partial dependence plots of the three variables showing the highest relative contribution scores within *B*. *bufo* models resulting from the three modelling approaches tested. On x-axis are shown the values of the predictors, and their units of measurement are indicated below the plots; on y-axis is reported the relative likelihood of occurrence.

**Fig 6:**
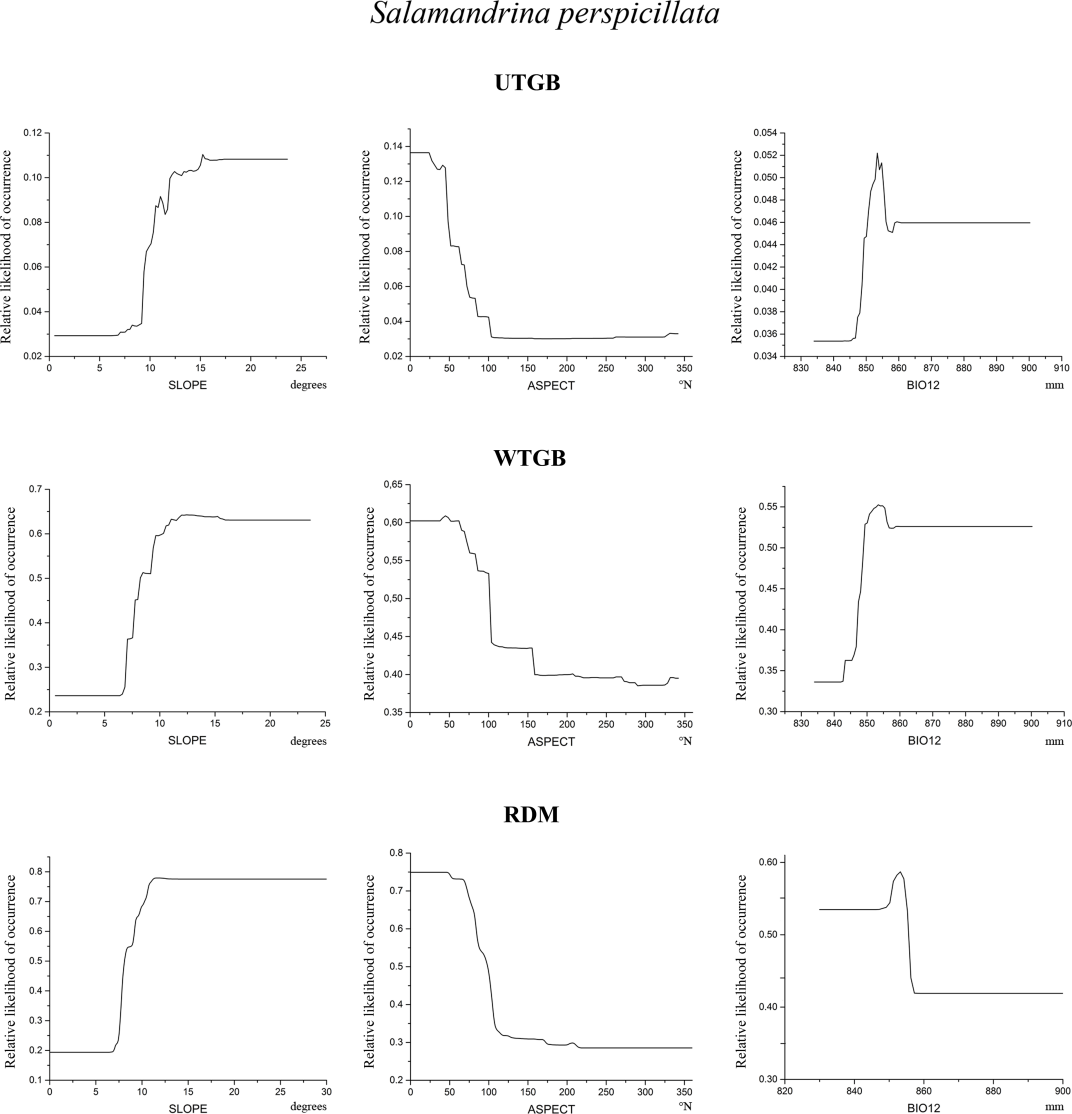
Partial dependence plots for *S*. *perspicillata*. Partial dependence plots of the three variables showing the highest relative contribution scores within *S*. *perspicillata* models resulting from the three modelling approaches tested. On x-axis are shown the values of the predictors, and their units of measurement are indicated below the plots; on y-axis is reported the relative likelihood of occurrence.

**Fig 7:**
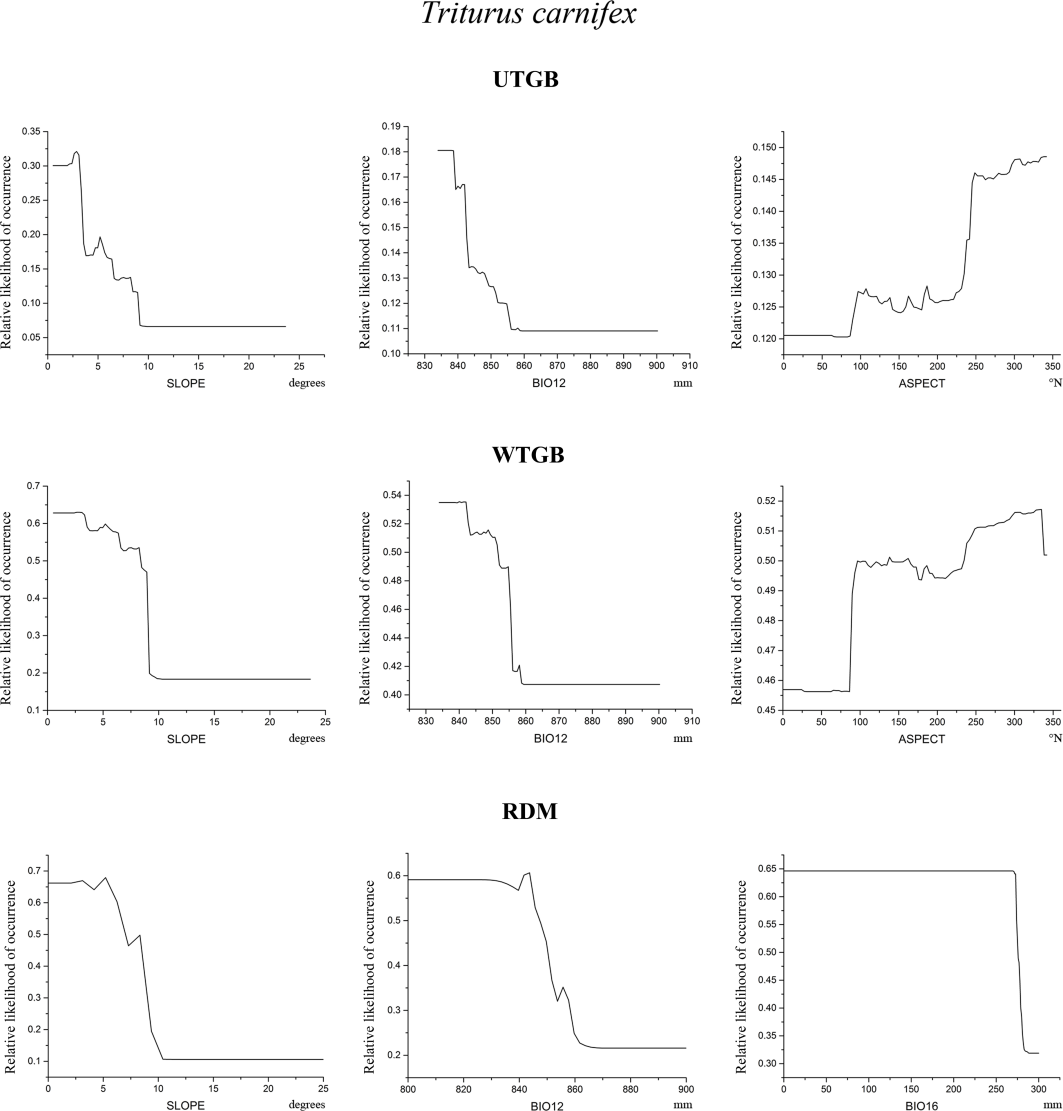
Partial dependence plots for *T*. *carnifex*. Partial dependence plots of the three variables showing the highest relative contribution scores within *T*. *carnifex* models resulting from the three modelling approaches tested. On x-axis are shown the values of the predictors, and their units of measurement are indicated below the plots; on y-axis is reported the relative likelihood of occurrence.

Figs 5–7 show, for each target species and for each modelling approach, the partial dependence plots, which report the variation of relative likelihood of occurrence consequent upon the variation in the predictors indicated in Table 5.

Some interesting results emerge from Fig 6: all the three models return the same pattern of relative likelihood of occurrence for the topographic variables SLOPE and ASPECT for *S*. *perspicillata*, whose curves follow a common trend. In fact, relative likelihood raises with increasing values of SLOPE and conversely it is higher for low values of ASPECT (i.e. north-east facing exposure). Also the three models obtained for *T*. *carnifex* (Fig 7) show a similar trend of relative likelihood of occurrence considering the predictor SLOPE. Relative likelihood tends to be higher for low values of this predictor, and then decreases rapidly towards low likelihood values as SLOPE increases. The only case in which the models obtained do not seem to define a clear pattern is for *B*. *bufo*. ASPECT is the first contributing variable for all the models, but with a different variation trend for the relative likelihood of occurrence in RDM compared to UTGB and WTGB (Fig 5). On the contrary, the plots for BIO8 resulting from UTGB and WTGB show a similar shape. The other most contributing variables for *B*. *bufo* vary among the three approaches tested.

Modelled pairwise interaction size for each target species and for each of the three approaches compared are reported in Table 6. These interactions were calculated for each of the possible pairs which can be formed within the set of the three most contributing variables emerging from the obtained BRT models (see Table 5).

**Table 6:** Pairwise interaction size for the three species and three methods tested.

Species	Pairwise interaction size					
	UTGB		WTGB		Averaged RDM	
	Variables	Score	Variables	Score	Variables	Score
*Bufo bufo*	ASPECT-BIO8	2.72	ASPECT-BIO8	1.72	BIO13-BIO17	4.68
	BIO14-BIO8	1.31	ASPECT-BIO4	0.20	ASPECT-BIO17	2.59
	ASPECT-BIO14	0.41	BIO8-BIO4	0	ASPECT-BIO13	2.21
*Salamandrina perspicillata*	SLOPE-ASPECT	26.17	SLOPE-BIO12	15.97	SLOPE-ASPECT	12.50
	ASPECT-BIO12	16.08	ASPECT-BIO12	10.07	SLOPE-BIO12	1.18
	SLOPE-BIO12	4.64	SLOPE-ASPECT	7.29	ASPECT-BIO12	0.71
*Triturus carnifex*	SLOPE-BIO12	3.96	SLOPE-BIO12	21.31	SLOPE-BIO16	10.96
	ASPECT-SLOPE	0.58	SLOPE-ASPECT	0.89	SLOPE-BIO12	8.55
	BIO12-ASPECT	0.13	ASPECT-BIO12	0.80	BIO12-BIO16	0.45

Pairwise interaction scores, for each target species and each modelling approach, within each of the possible pairs formed by the three variables showing the highest relative contribution scores in the corresponding BRT models.

**Fig 8:**
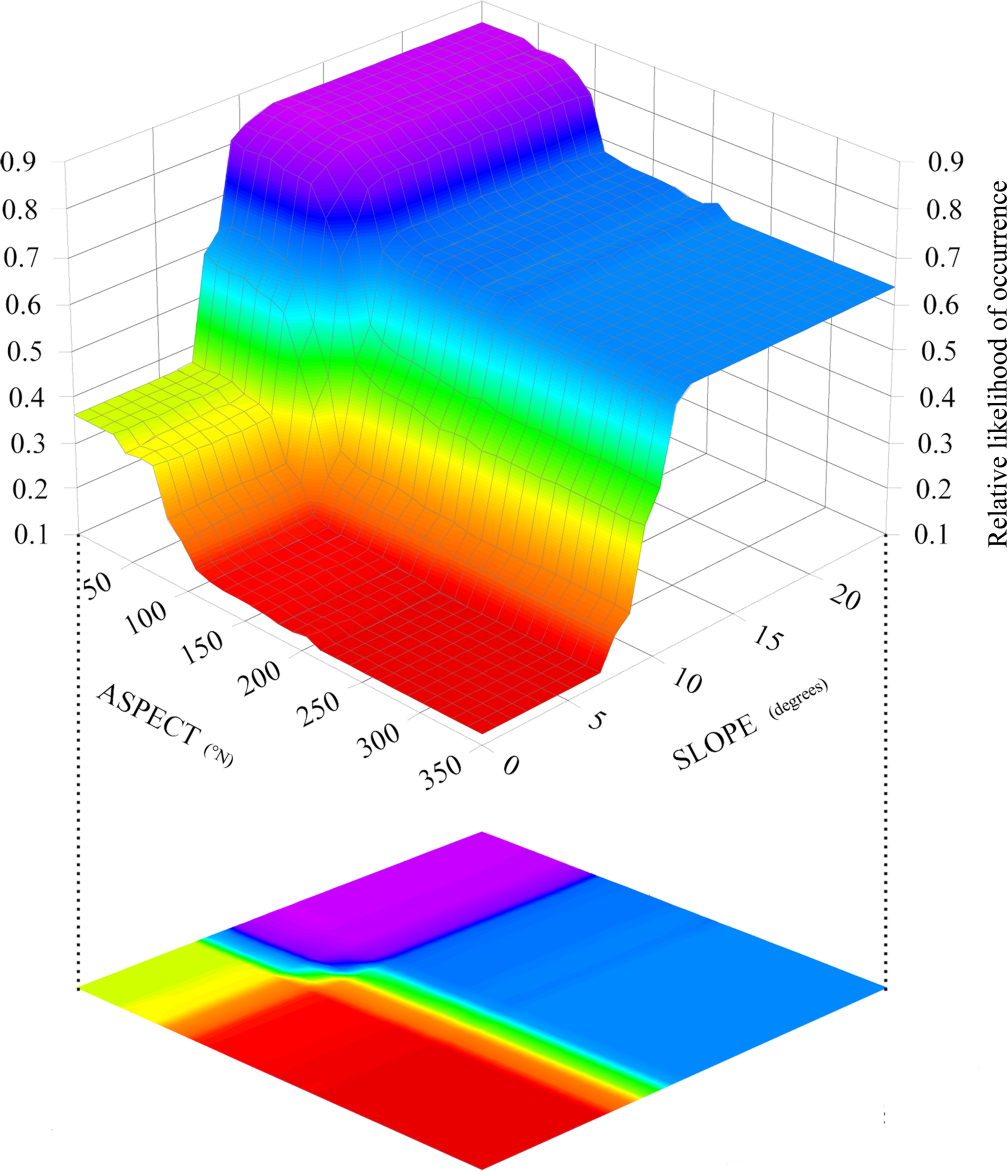
Pairwise interaction size. Plotted pairwise interaction between SLOPE and ASPECT resulting from the averaged RDM model for *S*. *perspicillata*. Areas in blue-violet represent the combined range of values for which relative likelihood of occurrence is higher.

There seem to be few strong pairwise interactions modelled within the three approaches compared. Nevertheless, Fig 8 shows the three-dimensional partial dependence plot resulting from the pair SLOPE-ASPECT within the averaged RDM for *S*. *perspicillata* as an example of additional information which could be extrapolated for conservation purposes from the assessment of the predictors interactions. It is visible from the 3D plot that a combination of north-eastern exposure and slope steeper than 10 degrees represents the environmental conditions corresponding to the highest relative likelihood of occurrence for this species.

## Discussion

Implementations of SDMs driven by a careful selection of the modelling techniques, with the best trade-off between the costs of setting up an appropriate database and the robustness of model predictions, may optimize information obtainable from the available data, thus improving the conservation planning process. In this context, the results presented in this paper provide useful information on which of the three pseudo-absence generation approaches considered (UTGB, WTGB and RDM) is more appropriate to model the potential distribution of species having different ecological and distributional characteristics, and needing conservation measures.

First, the RDM approach significantly ameliorates AUC scores for *B*. *bufo* and *T*. *carnifex* with respect to UTGB and WTGB, and the increased model discrimination within RDM is indicated also by the higher TSS with both ‘TSS max’ and ‘TSS 10^th^ percentile’ thresholds. Contrarily, the models built for *S*. *perspicillata* do not show significant differences among the three pseudo-absence generation approaches neither with regard to AUC nor considering TSS. Such differences may be ascribed to the distributional and ecological characteristics of the three target species; *B*. *bufo* and *T*. *carnifex* are widely distributed species [34, 36], while *S*. *perspicillata* shows stricter habitat requirements [35] resulting in a narrower potential distribution, as emerges also from all the BRT models obtained for this species (see Fig 4). Thus, for *S*. *perspicillata* both UTGB and RDM would be probably environmentally distant from presence sites [48], facilitating the discrimination task of the model and consequently leading to high discrimination metrics scores for all the three approaches tested. Differently, for *B*. *bufo* and *T*. *carnifex* the increase of model discrimination within the RDM approach may be due to the fact that the probability of selecting pseudo-absence sites environmentally distant from the occurrence localities would be greater when drawing pseudo-absences randomly than when considering as pseudo-absences the presence points of the other species included in the target group, since sites suitable for the presence of other amphibians may have environmental characteristics which would permit also the presence of these two species having broader environmental tolerance. These results are also coherent with previous studies reporting that SDMs built for generalist species tend to show worse discrimination than those for species which are more restricted in environmental and geographic space [17, 48, 55].

Contrarily to what emerges for model discrimination, the results of the ‘inter-model’ two-tailed *t*-tests performed for *B*. *bufo* and of the ‘inter-model’ Wilcoxon-Mann-Whitney tests performed for *S*. *perspicillata* and *T*. *carnifex*, coupled with the boxplots shown in Fig 3, clearly indicate that the WTGB approach significantly ameliorates model calibration with respect to UTGB and RDM for all the three target species. Moreover, the absence of significant differences in model discrimination between the UTGB and WTGB approaches agrees with results presented in the Appendix S5 of Elith and Graham [41], indicating that balancing the weights of presences and pseudo-absences has no effect on discrimination capability of BRT models, as long as the selection of the most influential variables is not modified in the weighted model with respect to the unweighted one.

Thus, the weighting scheme balancing presences and pseudo-absences emerges as particularly suitable to optimize calibration when using target-group-based pseudo-absences, both for widespread species (as *B*. *bufo*) and species which are restricted in geographical and/or environmental space (as *S*. *perspicillata*).

Further interesting information emerging from Fig 3 refers to the much larger range of deviance within the RDM models obtained for each species with respect to the range of deviance within the corresponding UTGB and WTGB models; this pattern highlights the higher degree of uncertainty intrinsic to the generation of randomly drawn pseudo-absences.

Considering the discretized areas of relative likelihood of occurrence predicted by UTGB and WTGB, it clearly emerges a more conservative pattern for the UTGB (predominance of predictions in the lower classes) than for the WTGB approach. This finding corroborates what stated in Elith and Graham [41], i.e. the fact that an unweighted model with many more pseudo-absences than the available presences produces predictions showing predominantly low values, while the application of weights on pseudo-absences produces predictions more equally distributed across the response range.

The predictions obtained from the averaged RDM models show a further shift towards central and higher classes of relative likelihood of occurrence, covering the whole response range. Even though this trend does not provide information about the RDM predictive accuracy, the fact that it emerged for all the target species (ranging in a “steno-to-euryecious” habit gradient) may suggest that RDM suffers from a “niche-width and prevalence effect” less than the others two modelling approaches. Nevertheless, the relative likelihood of occurrence resulting from the RDM models is not always shifted towards higher classes; in fact, the predominance of the central class (0.4–0.6) found in *B*. *bufo* seems to actually reflect the distributional characteristics of a generalist species, as *B*. *bufo* is [34].

The closeness among the relative contribution scores of the three most influential predictors (i.e. no apparent highly discriminant variable) for *B*. *bufo* within each of the three modelling approaches further suggests the euryecious habits of this species, whereas larger gaps emerged for *S*. *perspicillata* and *T*. *carnifex*. In fact, SLOPE is the most contributing variable within all the models obtained for these two species, with ASPECT and the annual precipitation (BIO12) interchanging each other as the second and third most influential predictors. Interestingly, large gaps in the relative contribution score between the most contributing predictor and the second and third emerged in particular from WTGB models with respect to UTGB. This finding suggests that when many more pseudo-absences than presences are generated, a weighted scheme permits to more easily identify the predictors which most shape the species distribution. This further confirms that the weighted scheme improves the use of target-group-based pseudo-absences, since it ameliorates both calibration and isolation of highly contributing variables.

The high contribution of SLOPE for *S*. *perspicillata* in all the three modelling approaches matches previous observations by other authors [56], who suggest a connection between slopes and presence of rocks and logged trees, used by this species as a daylight refugia with anti-predatory function. Results obtained for ASPECT also agree with previous researches [57], confirming the trend of *S*. *perspicillata* to live in cool and wet environments with north exposure.

On the other hand, *T*. *carnifex* is usually found in ponds and creeks’ bends [58], which are environments usually requiring none or moderate slope, thus explaining the strong contribution of this variable and the corresponding trend of relative likelihood of occurrence found in all the models built.

With regard to *B*. *bufo*, the lack of consensus on the most contributing predictors among the three modelling approaches may be due, as already suggested above, to the ubiquitous attitudes of this species [59, 60], which is known to be the most euryecious amphibian in Europe [58, 61], and to the consequent difficulty of modelling the variables effect on relative likelihood of occurrence. Moreover, many presence points may be biased by migrating adults or juvenile dispersal, or by occasional reports in urban environments. As a consequence, the “valleys” and “peaks” showed by some of the partial dependence plots obtained for *B*. *bufo*, rather than representing actual effects of the predictors, could be the result of how data are distributed within the predictors range of values. In fact, when only few data fall in some portions of a predictor range, BRT might overfit the response to the available sample, thus producing confounding effects [41].

Finally, specific attention should be paid to pairwise interactions: since they efficiently represent the effect of two variables interacting each other, the results obtained in our case-study may have great implications in conservation and PAs’ planning. A clear example comes from the useful information provided for *S*. *perspicillata*, for which high values of slope, combined with a certain range of orientation, produced higher relative likelihood of occurrence (see Fig 8) than these two variables can produce alone. It is therefore presumable that territories with north-eastern aspect and with high degrees of slope are particularly suitable for *S*. *perspicillata*, and therefore may be prioritised in the context of biodiversity conservation and/or PAs management plans.

## Conclusions

PAs are spatially defined entities aiming to preserve a certain arrangement of biotic elements, whose accurate knowledge is fundamental to correctly manage the PAs themselves. The application of ecological modelling to investigate the distribution of such biotic elements needs fine-scale data, in order to better describe the specific relationships between the target species and the range of environments characterising a certain area.

Results of the present work suggest some useful guidelines to efficiently apply the BRT modelling technique within conservation studies based on presence-background data sets. First, when occurrence data for a set of species assumed suitable as target-group are available, the WTGB approach represents an appropriate choice to optimize model performance with regard to calibration and identification of highly contributing predictors. Nevertheless, since structured data sets comprising presence records for an entire target-group are rarely available, the use of RDM approach could be an adoptable alternative when only presence records for the target species are available. In fact, models built through the RDM approach showed good discrimination power, highlighted by both AUC and TSS scores, and the capability of clearly isolate highly contributing variables from the set of input predictors. Still, a clear calibration deficit with respect to the UTGB and WTGB models built for the same species resulted as the main flaw of the RDM. When using this approach, we recommend, following Barbet-Massin, Jiguet [20], the generation of a number of random pseudo-absences close to that of the available presence points, since in this way the resulting modelled distribution would reflect the areas where the target species is highly likely to occur (i.e. maximising specificity); this is an appropriate result for conservation studies.

Results obtained for *B*. *bufo* from all the three pseudo-absences generation approaches tested, with respect to both discrimination and calibration, confirmed that modelling the distribution of generalist species is a difficult task even when using robust modelling techniques. So, when dealing with such species it is particularly important both to verify the way occurrence records were collected and to carefully interpret model predictions, always keeping in mind that a consistent and homogeneous set of carefully assessed presence points provides greater meaningfulness to any pseudo-absences generation technique.

## Supporting information

S1 Dataset Dataset of the presence and pseudo-absence points for the three target species. Zip archive with.csv files, one for each target species, reporting the coordinates of the respective presence and target-group-based pseudo-absence points. Coordinates are in decimal degrees, UTM-WGS84 reference system.
(ZIP)

S1 Fig Modelled distribution of all the target species for each of the four replicates of RDM approach. Maps, for all the species considered, resulting from each of the 4 replicates of 10-fold cross-validated BRT model built on pseudo-absences drawn at random excluding presence localities (RDM). The first, second, third and fourth replicate for each species are indicated, respectively, with r_1_, r_2_, r_3_ and r_4_.
(TIF)

S2 Fig True Skill Statistic (TSS) plots for UTGB and WTGB approaches. Plots showing the variation of the True Skill Statistic (TSS) resulting from the UTGB and WTGB, as a function of increasing threshold values. Curves for each species result from the 10-fold cross-validation process used in the two BRT approaches.
(TIF)

S3 Fig True Skill Statistic (TSS) plots for RDM approach (left panel: average, right panel: all replicates). Plots showing the variation of the True Skill Statistic (TSS) resulting from the RDM, as a function of increasing threshold values. On the left panel are shown the curves resulting from the averaged 4-replicates, 10-fold cross-validated RDM models, while in the right panel are shown, with the same color legend for the three species, the curves for each of the four replicates.
(TIF)
