# Supplementary figures and images for: Comparing pseudo-absences generation techniques in Boosted Regression Trees models for conservation purposes: A case study on amphibians in a protected area
Source: PLoS One. 2017 Nov 6;12(11):e0187589. doi: 10.1371/journal.pone.0187589 (PMC5673221; doi:10.1371/journal.pone.0187589)

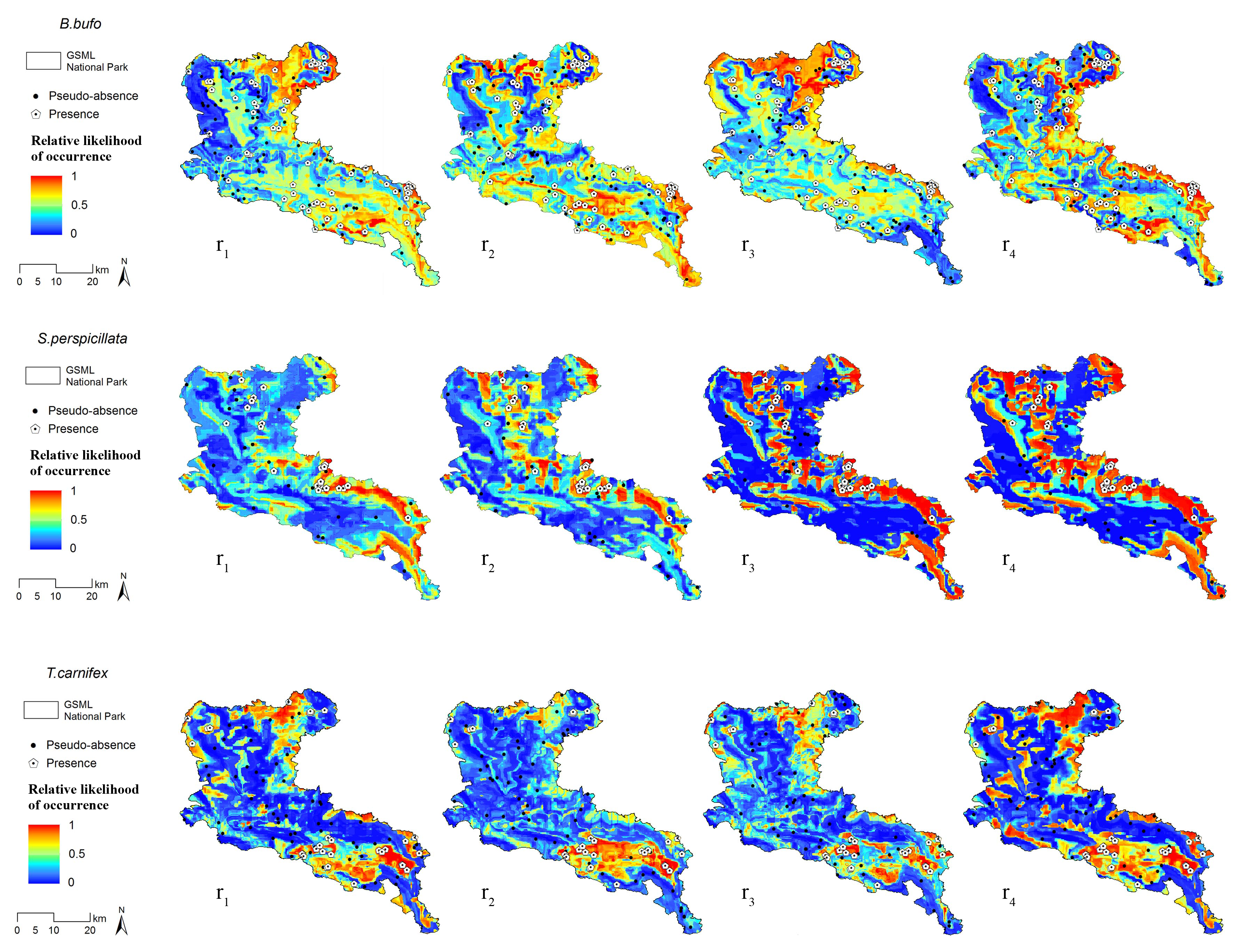

Supplement: S1 Fig — Maps, for all the species considered, resulting from each of the 4 replicates of 10-fold cross-validated BRT model built on pseudo-absences drawn at random excluding presence localities (RDM). The first, second, third and fourth replicate for each species are indicated, respectively, with r1, r2, r3 and r4. (TIF) [file pone.0187589.s002.tif]

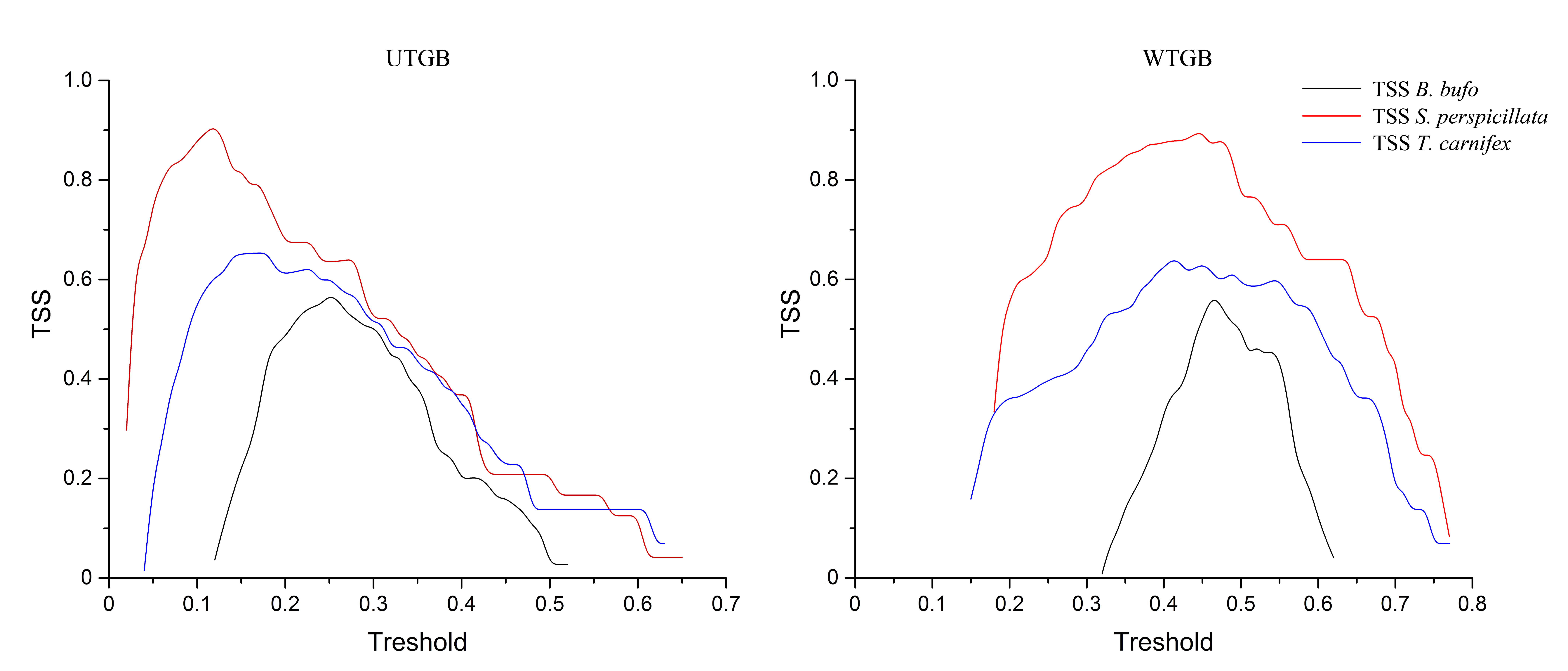

Supplement: S2 Fig — Plots showing the variation of the True Skill Statistic (TSS) resulting from the UTGB and WTGB, as a function of increasing threshold values. Curves for each species result from the 10-fold cross-validation process used in the two BRT approaches. (TIF) [file pone.0187589.s003.tif]

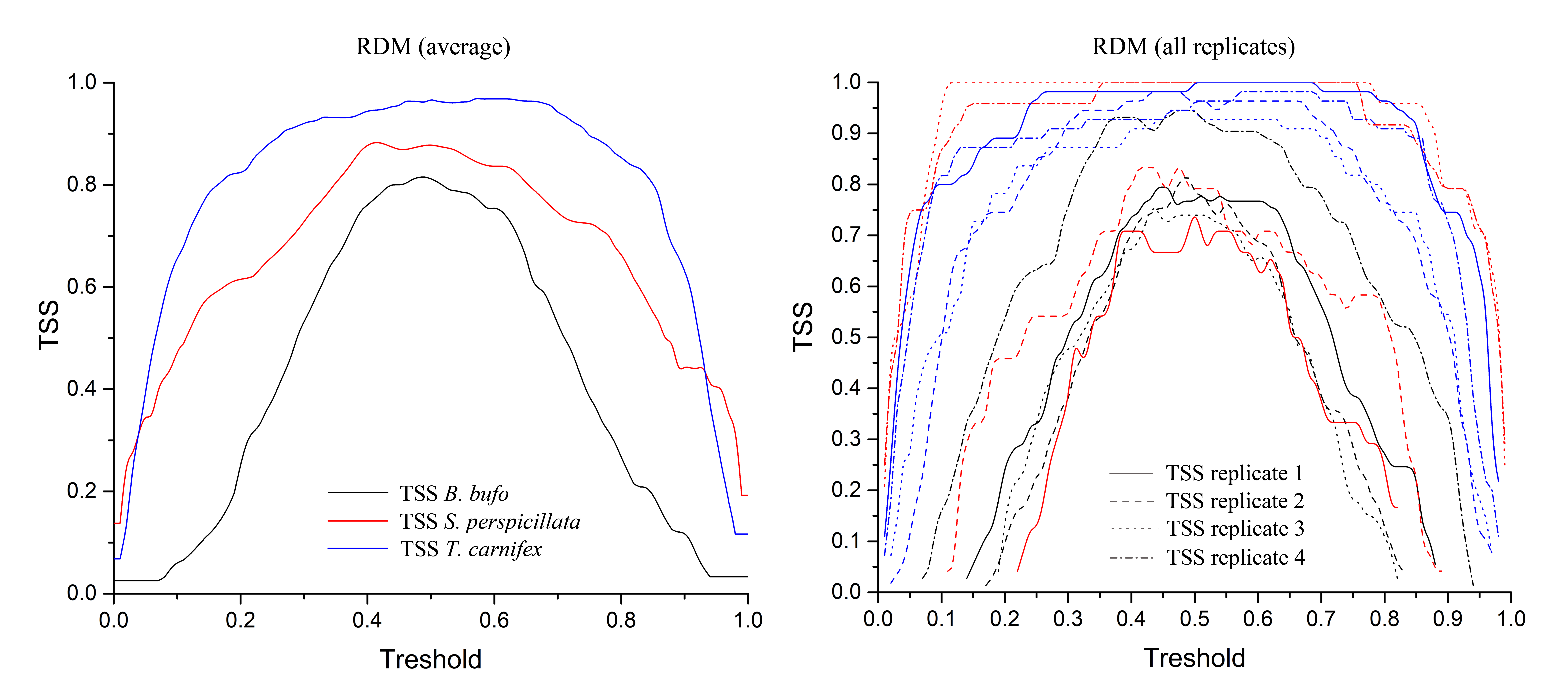

Supplement: S3 Fig — Plots showing the variation of the True Skill Statistic (TSS) resulting from the RDM, as a function of increasing threshold values. On the left panel are shown the curves resulting from the averaged 4-replicates, 10-fold cross-validated RDM models, while in the right panel are shown, with the same color legend for the three species, the curves for each of the four replicates. (TIF) [file pone.0187589.s004.tif]
